# HbA1c comparable to fasting glucose in the external validation of the African Diabetes Risk Score and other established risk prediction models in Black South Africans

**DOI:** 10.1186/s12902-024-01735-w

**Published:** 2024-10-10

**Authors:** Nicola Royce, Héléne T Cronjé, André P Kengne, Herculina S Kruger, Robin C Dolman-Macleod, Marlien Pieters

**Affiliations:** 1 Centre of Excellence for Nutrition, Faculty of Health Sciences, North-West University, Potchefstroom Campus Private Bag X6001, Potchefstroom, 2520 South Africa; 2 Department of Public Health, Section of Epidemiology, University of Copenhagen, Copenhagen, Denmark; 3 Non-Communicable Diseases Research Unit, South African Medical Research Council, Cape Town, South Africa; 4 Department of Medicine, University of Cape Town, Cape Town, South Africa; 5 SAMRC Extramural Unit for Hypertension and Cardiovascular Disease, Faculty of Health Sciences, North-West University, Potchefstroom, South Africa

**Keywords:** Type 2 diabetes, African, Epidemiology, Diabetes risk score, Validation

## Abstract

**Background:**

The use of non-invasive risk scores to detect undiagnosed type 2 diabetes (T2D) ensures the restriction of invasive and costly blood tests to those most likely to be diagnosed with the disease. This study assessed and compared the performance of the African Diabetes Risk Score (ADRS) with three other diabetes risk prediction models for identifying screen-detected diabetes based on fasting plasma glucose (FPG) or glycated haemoglobin (HBA1c).

**Methods:**

Age, sex, waist circumference, body mass index, blood pressure, history of diabetes and physical activity levels from the SA-NW-PURE study were used to externally validate the ADRS and other established risk prediction models. Discrimination was assessed and compared using C-statistics and nonparametric methods. Calibration was assessed using calibration plots, before and after recalibration.

**Results:**

Nine hundred and thirty-seven participants were included; 14% had prevalent undiagnosed T2D according to FPG and 26% according to HbA1c. Discrimination was acceptable and was mostly similar between models for both diagnostic measures. The C-statistics for diagnosis by FPG ranged from 0.69 for the Simplified FINDRISC model to 0.77 for the ADRS model and 0.77 for the Simplified FINDRISC model to 0.79 for the ADRS model for diagnosis by HbA1c. Calibration ranged from acceptable to good, though over- and underestimation were present. All models improved significantly following recalibration.

**Conclusions:**

The models performed comparably, with the ADRS offering a non-invasive way to identify up to 79% of cases. Based on its ease of use and performance, the ADRS is recommended for screening for T2D in certain Black population groups in South Africa. HbA1c as a means of diagnosis also showed comparable performance with FPG. Therefore, further validation studies can potentially use HbA1c as the standard to compare to.

**Supplementary Information:**

The online version contains supplementary material available at 10.1186/s12902-024-01735-w.

## Introduction

Diabetes is a growing problem worldwide. Type 2 diabetes (T2D) and intermediate hyperglycaemia are becoming increasingly burdensome to residents and healthcare systems in low- and middle-income countries (LMICs), even though the majority of cases remain undiagnosed [1]. Africa is expected to experience the greatest increase in diabetes prevalence by 2045 [2]. These patterns are also observed in South Africa, where the T2D epidemic occurs alongside the communicable disease epidemic of human immunodeficiency virus and tuberculosis [3]. This strains the public healthcare system, which services over 80% of the population [4].

In South Africa, 45% of the estimated T2D cases are undiagnosed [2]. Hyperglycaemia results in pathological and functional changes and may persist for an extended period before being diagnosed [5]. The chronic nature of T2D requires continuous clinical care and management that requires significant healthcare resources. In South Africa, the direct cost of managing T2D amounted to over ZAR 2.7 billion in 2018 (approximately US$143 million), half of which related to treating complications. This cost is expected to increase at least 10-fold over the next decade [6]. Detecting T2D early in the pathophysiological process enables early administration of patient-centred management to optimise glycaemia and minimise complications, subsequently reducing its associated health and economic burden [7–9].

As glucose testing cannot be applied broadly in resource-poor settings such as South Africa, a risk assessment score based on non-invasive predictors is a quick method of guiding healthcare professionals as to whether a blood-based diagnostic test should be performed [10]. This ensures the allocation of scarce resources to those at highest risk to prevent the progression of T2D and associated complications [11]. Risk scores can also be used as health promotion tools in LMIC settings when used as self-completion questionnaires. The information provided at the end of the questionnaire, based on the risk status, can potentially act as a catalyst for needed lifestyle changes [12].

Several non-invasive risk assessment tools have been developed in specific White, multi-racial, Asian, and Middle Eastern cohorts, which limits their generalisability to other populations, such as individuals of African descent [13–16]. Prediction equations developed in other population groups that only include non-invasive variables have performed poorly in Black African cohorts, and either underestimated or overestimated T2D risk [14, 16]. This is because there are different thresholds, for example, for age, body mass index (BMI), and waist circumference in terms of T2D risk in Africans compared to individuals of European descent [16]. To date, only one sub-Saharan African screening tool has been developed using data from Tanzania, Senegal, and Guinea, known as the African Diabetes Risk Score (ADRS). The predictive accuracy for screen-detected T2D of this screening tool was found to be good in two Black cohorts from Cape Town (CRIBSA Study) and rural Kwa-Zulu Natal [17]. As ethnicity influences T2D risk [10], the ADRS requires further validation in other ethnic groups from a variety of settings in South Africa to ensure that it reliably predicts the risk of undiagnosed T2D in Black individuals with different demographic and ethnic characteristics.

This study aimed to further validate the ADRS in the South African North-West Province arm of the Prospective Rural and Urban Epidemiology (SA-NW-PURE) study. The American Diabetes Association (ADA) risk score, a Simplified Finnish Diabetes Risk Score (FINDRISC), and the Indian Risk Score (IRS), which were developed in different phenotypic and racial populations, were also validated to compare the predictive performance of the ADRS [13, 18, 19]. Furthermore, this study evaluated whether predictive performance differed between fasting plasma glucose (FPG)-based or glycated haemoglobin (HBA1c)-based diagnoses and across age, BMI, sex, and rural-urban living subgroups.

## Materials and methods

### Study population and design

The PURE study is a large-scale epidemiological study that prospectively investigates lifestyle behaviours, cardiovascular risk factors and chronic non-communicable disease incidence among different communities across 27 low-, middle-, and high-income countries [20]. This study used data from SA-NW-PURE, collected at three time points (2005, 2010 and 2015). Self-reported Tswana speaking, apparently healthy Black adults older than 30 years of age were eligible for inclusion at baseline (2005). Any self-reported prior cardiovascular event or acute illness was basis for exclusion. Detailed information on participant selection and recruitment has been reported previously [20, 21]. The SA-NW-PURE study included two dwelling sites (urban and rural) and consisted of *n* = 2 010 participants at baseline. In 2010, *n* = 1 282 participants returned for follow-up, and *n* = 924 returned in 2015. All participants provided written informed consent prior to data collection. The study received ethical approval from the North-West University Health Research Ethics Committee (NWU-00190-22-A1) and complies with the amended Declaration of Helsinki.

Newly diagnosed T2D was defined as having FPG levels  ≥ 7 mmol/L or HbA1c  ≥ 6.5%, in line with current guidelines [22], at any of the three data collection time-points. These participants (cases) were investigated alongside individuals for whom FPG or HbA1c were below the established cut-offs throughout follow-up (participants without T2D). Individuals who were receiving diabetes medication at any time point were excluded. Data collected at the first time of diagnosis for participants with T2D (this could be either time point 1, 2 or 3), and at baseline for participants without T2D, were used in the risk prediction models. In all, *n* = 937 participants were included in this cross-sectional analysis. Supplemental Fig. 1 provides an overview of the case-status across data collection time points.

### Data collection

Information regarding general demographics, tobacco use, alcohol consumption, dietary intake, physical activity, and medicine use (including lipid-lowering, antihypertensive, and glucose-lowering medication) was collected by trained field workers using standardised, validated questionnaires. Weight, height, and waist circumference were measured according to the International Standards of Anthropometric Assessment (ISAK). BMI was calculated by dividing weight by height squared and reported as kg/m^2^. Blood pressure was measured in mmHg using the OMRON HEM-757 (Omron Healthcare, Kyoto, Japan) automated digital blood pressure monitor in 2005 and 2010, and the OMRON M6 device (Omron Healthcare, Kyoto, Japan) in 2015. Fasting (overnight) blood samples were collected, processed, and stored at − 80 °C until analyses. Samples for FPG were collected in fluoride tubes, and FPG was quantified using an enzymatic reference method with hexokinase on a Vitros DT6011 Chemistry Analyzer (Ortho-Clinical Diagnostics, Rochester, New York, USA) in 2005, and a Cobas Integra 400 Roche Clinical System (Roche Diagnostics, Indianapolis, IN, USA) in 2010 and 2015. Samples for glycated haemoglobin (HbA1c) were collected in EDTA tubes and determined via ion exchange high-performance liquid chromatography with the D-10 Haemoglobin testing system at all time points (Biorad, Hercules, California, USA).

### Risk prediction models

The ADRS is the only prediction model that has been developed in a sub-Saharan African population [17]. The FINDRISC model is a widely used diabetes risk score that was developed in a White population [23] and subsequently broadly validated [24, 25]. Physical activity and diet were excluded in the Simplified FINDRISC, a derivative of the FINDRISC model developed by Bergmann et al. (2007), as these variables were found to have limited relevance [23]. The ADA risk model was developed in a multi-ethnic population and is used on a large scale in the United States [13]. The IRS model was developed in an Indian population [19]. Details of the different risk prediction models are provided in Supplemental Table 1.

### Statistical analysis

The participant distribution (T2D vs. no T2D) based on FPG and HbA1c diagnosis is shown in Supplemental Table 2. In terms of precision, Vergouwe et al. [26] suggest that a minimum of 100 events (T2D) and 100 non-events are required for the validation of binary outcomes. In the SA-NW-PURE study, at least 100 people were diagnosed with T2D in total using either FPG or HbA1c. The predicted probability of undiagnosed T2D for each participant was estimated using the relevant predictors for each model [13, 17–19]. Exclusions based on missing data were model-specific, resulting in differential cohort sizes across models. Model performance was assessed using discrimination and calibration statistics.

Discrimination refers to the ability of the model to distinguish those with prevalent undiagnosed T2D from those without T2D. Discrimination was assessed and compared using the concordance (C) statistic and non-parametric methods [27, 28]. C-statistics vary from no discrimination (0.5) to perfect discrimination (1.0), where values of 0.6–0.7 are deemed to be poor, 0.7–0.8, acceptable and 0.8–0.9, good [29]. Another popular method for displaying the discriminatory accuracy of a potential novel marker (diagnostic test), is the receiver operating characteristic (ROC) curve [30]. ROC curves are used to calculate the Youden Index, which is the optimal cut-off for maximising the potential effectiveness of a model, where sensitivity and specificity are determined for each threshold [31]. This index ranges between 0 and 1, where 1 indicates complete separation between diseased and healthy populations and 0 indicates complete overlap [30].

Calibration refers to the agreement between the probability of the outcome of interest as estimated by the model and the observed outcome frequencies [32]. This was assessed by plotting the predicted risk against the observed outcome rate in calibration plots, as well as utilising the Hosmer and Lemeshow goodness-of-fit test [29, 33]. Furthermore, the agreement between the expected and observed T2D rates (E/O) was assessed, where the 95% confidence intervals (CIs) were calculated assuming a Poisson distribution [33]. Ideally, the E/O rate should be as close to 1 as possible, values below 1 underestimate, and values above 1 overestimate the risk of undiagnosed T2D prevalence.

The Yates slope and the Brier score were also calculated. The Yates slope is the difference between the mean predicted probability of T2D for participants with and without prevalent T2D, where higher values indicate better performance [30]. The Brier score is the squared difference between the predicted probability and the actual outcome for each participant, where a perfect prediction model has a value of 1, and 0 indicates no match in prediction and outcome [32, 34].

To reduce bias brought on by differences in T2D prevalence between the development and test (validation) populations, all models were recalibrated according to the SA-NW-PURE-specific T2D prevalence using intercept adjustment. The calculated correction factor is based on the mean predicted risk and the prevalence in the SA-NW-PURE dataset and is the natural logarithm of the odds ratio of the mean observed prevalence and the mean predicted risk [35]. Furthermore, sensitivity analyses were conducted to assess the aforementioned aspects of model performance using first FPG and then HbA1c for T2D diagnosis.

Data analysis used R^®^ statistical software version 4.1.1 (2021), and the level of statistical significance was set at *p* < 0.05. The models were validated in the overall sample and compared between sex (men vs. women), median age (< 51 years vs.  ≥ 51 years), median BMI (< 25 vs. ≥  25 kg/m^2^), and residency (rural vs. urban) subgroups upon recalibration.

## Results

### Participant characteristics

The prevalence of T2D, according to FPG, was approximately 14%, whereas it was just over 26%, according to HbA1c (Supplemental Table 2). Supplemental Table 3 provides the descriptive characteristics of the full analytical cohort (*n* = 937), and across the four case–non-case groups. More women were diagnosed with T2D by HbA1c than FPG (79.8% vs. 69.6%). Overall, those with T2D were older, had higher waist circumferences, and were more likely to be women, overweight or obese, and hypertensive. Very few participants with T2D reported having a family history of diabetes, although this data should be interpreted cautiously as almost 40% of the participants had missing family history data.

### Prediction of prevalent undiagnosed T2D

The number of variables included in the risk prediction models ranged from three (ADRS) to six (ADA). All included age and waist circumference, and hypertension status was only excluded by the IRS. The Simplified FINDRISC model included characteristics with the least amount of missing data and thus had the largest sample size for analysis, whereas the ADA had the smallest sample size (Table 1).

**Table 1:** Performance of the original risk prediction models and the recalibrated models based on diagnosis by FPG and HbA1c

	ADRS		Simplified FINDRISC		ADA		IRS	
T2D Diagnosed by FPG								
Sample Size	*n* = 720 (100 cases)		*n* = 727 (101 cases)		*n* = 420 (49 cases)		*n* = 423 (49 cases)	
**Performance**	**Original**	**Recalibrated**	**Original**	**Recalibrated**	**Original**	**Recalibrated**	**Original**	**Recalibrated**
E/O [95% CI]	0.15 [0.12–0.18]	1.01 [0.83–1.23]	0.24 [0.20–0.30]	0.96 [0.79–1.17]	6.40 [5.27–7.78]	2.76 [2.27–3.35]	0.70 [0.57–0.85]	0.90 [0.74–1.09]
Brier score	0.13	0.11	0.13	0.12	0.67	0.19	0.10	0.10
Yates slope	0.02	0.11	0.02	0.08	0.11	0.27	0.05	0.07
C-statistic [95% CI]	0.77 [0.73–0.82]	-	0.69 [0.63–0.74]	-	0.75 [0.69–0.81]	-	0.74 [0.67–0.81]	-
Optimal threshold	0.01	0.08	0.02	0.09	0.92	0.21	0.11	0.15
Sensitivity	87.0	-	70.0	-	88.0	-	67.0	-
Specificity	55.0	-	66.0	-	51.0	-	71.0	-
**T2D Diagnosed by HbA1c**								
**Sample Size**	***n*** ** = 815 (215 cases)**		***n*** ** = 822 (218 cases)**		***n*** ** = 477 (124 cases)**		***n*** ** = 480 (125 cases)**	
**Performance**	**Original**	**Recalibrated**	**Original**	**Recalibrated**	**Original**	**Recalibrated**	**Original**	**Recalibrated**
E/O [95% CI]	0.08 [0.07–0.09]	0.84 [0.73–0.95]	0.13 [0.11–0.15]	0.81 [0.71–0.92]	3.35 [2.94–3.83]	1.93 [1.69–2.20]	0.37 [0.32–0.42]	0.96 [0.84–1.09]
Brier score	0.25	0.16	0.24	0.17	0.55	0.23	0.20	0.16
Yates slope	0.02	0.18	0.04	0.17	0.11	0.31	0.06	0.14
C-statistic [95% CI]	0.79 [0.75–0.82]	-	0.77 [0.73–0.80]	-	0.78 [0.73–0.82]	-	0.79 [0.75–0.83]	-
Optimal threshold	0.01	0.17	0.02	0.16	0.92	0.38	0.08	0.24
Sensitivity	80.0	-	74.0	-	89.0	-	78.0	-
Specificity	64.0	-	71.0	-	55.0	-	73.0	-

FPG – fasting plasma glucose; HbA1c – glycated haemoglobin; E/O – expected/observed

Sample sizes differ for each risk prediction model, as participants with missing data were excluded per model

### Discrimination

**Fig. 1:**
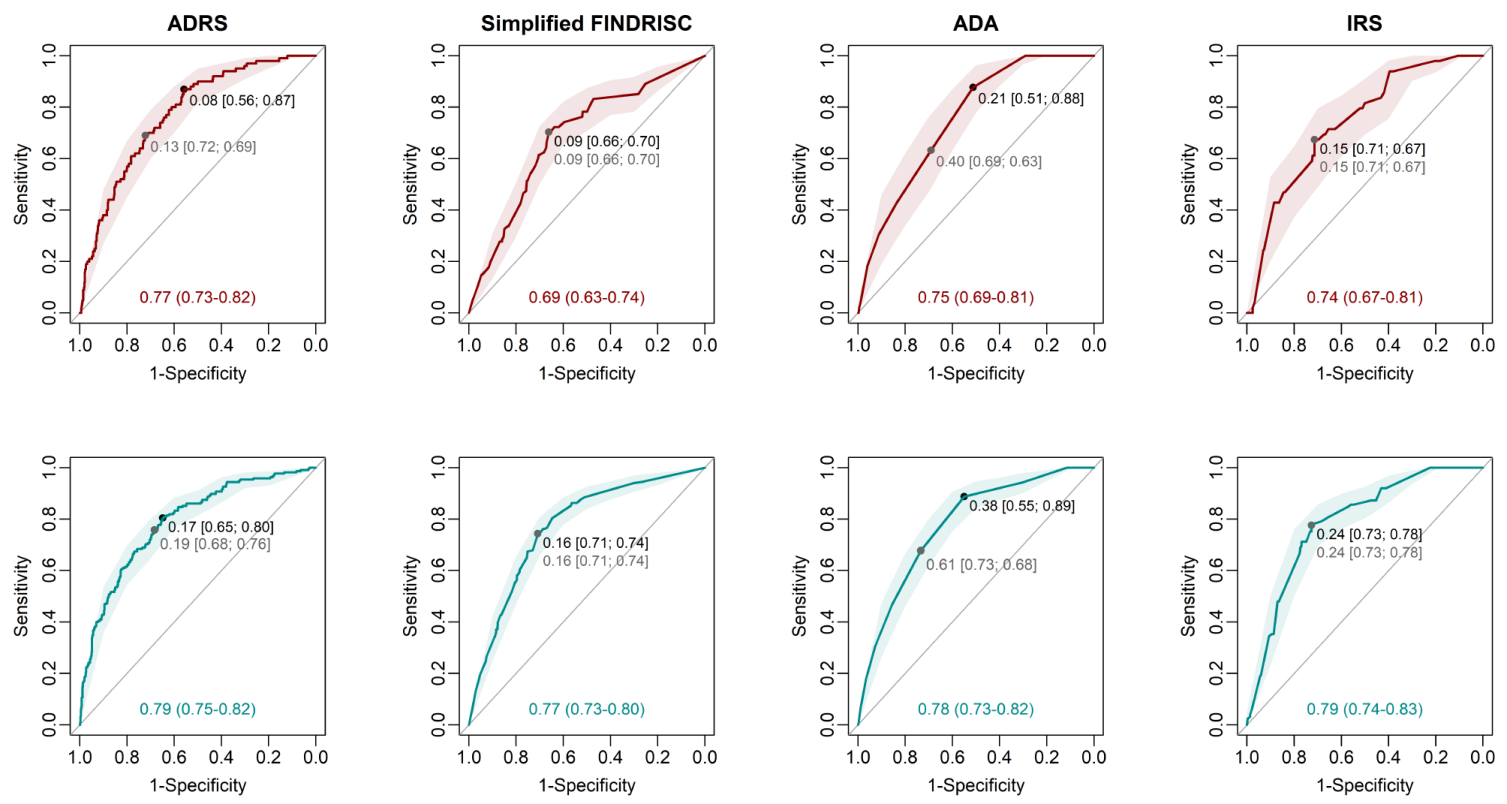
ROC curves for each risk prediction model by FPG-diagnosis (red) and HbA1c-diagnosis (blue), and their respective C-statistic and optimal threshold values from the Youden Index approach (black values) and the top left point approach (grey values). The numbers featured in the square brackets are co-ordinates

Table 1 includes the C-statistics and sensitivity and specificity measures at the optimal threshold, used to compare discrimination ability across models. Discrimination was acceptable overall and tended to be better in HbA1c- vs. FPG-based diagnoses. The ADRS had the highest C-statistic for T2D by FPG diagnosis and performed as well as the IRS for T2D diagnosis by HbA1c, whereas the Simplified FINDRISC performed the worst in both diagnostic groups. The ADA risk score was the most sensitive and least specific score in both diagnostic models, followed by the ADRS in each case. Overall, the IRS provided the best sensitivity/specificity profile (Fig. 1). When comparing C-statistics between subgroups (Table 2), the Simplified FINDRISC and IRS models performed better in participants below vs. above the median age for both, and there was better ADA risk score discrimination in rural- vs. urban-dwelling individuals and those with BMIs below vs. above the median. The ADRS performed equally well in all subgroups.

**Table 2:** Performance of the recalibrated risk prediction models according to sex, median age, median BMI, and residency based on diagnosis by FPG and HbA1c

Models		Men	Women	*P*-Value	Age < 51	Age ≥ 51	*P*-Value	BMI < 25 kg/m^2^	BMI ≥ 25 kg/m^2^	*P*-Value	Rural	Urban	*P*-Value
Sample Size		*n* = 261 (30 cases)	*n* = 676 (70 cases)		*n* = 447 (35 cases)	*n* = 490 (65 cases)		*n* = 477 (25 cases)	*N* = 460 (75 cases)		*n* = 527 (47 cases)	*n* = 410 (53 cases)	
**ADRS**													
FPG-based diagnosis	E/O	0.86 [0.60–1.24]	1.06 [0.84–1.34]		0.88 [0.63–1.22]	1.00 [0.79–1.28]		1.14 [0.77–1.69]	0.92 [0.73–1.15]		1.13 [0.85–1.51]	0.89 [0.68–1.16]	
	C-Statistic	0.77 [0.68–0.85]	0.78 [0.73–0.83]	0.86	0.78 [0.70–0.85]	0.73 [0.67–0.79]	0.34	0.77 [0.67–0.86]	0.67 [0.60–0.74]	0.10	0.78 [0.72–0.85]	0.76 [0.69–0.82]	0.59
HbA1c-based diagnosis	E/O	1.04 [0.78–1.40]	0.78 [0.67–0.91]		0.98 [0.76–1.28]	0.77 [0.66–0.90]		1.09 [0.82–1.43]	0.74 [0.63–0.86]		0.97 [0.79–1.18]	0.73 [0.61–0.87]	
	C-Statistic	0.76 [0.68–0.85]	0.97 [0.75–0.83]	0.50	0.78 [0.71–0.86]	0.72 [0.66–0.77]	0.13	0.67 [0.59–0.76]	0.72 [0.67–0.77]	0.34	0.76 [0.71–0.82]	0.80 [0.76–0.85]	0.25
**Sample Size**		***n*** **= 261** **(30 cases)**	***n*** **= 676** **(71 cases)**		***n*** **= 447** **(35 cases)**	***n*** **= 490** **(66 cases)**		***n*** **= 447** **(25 cases)**	*n* = 490 **(76 cases)**		***n*** **= 527** **(47 cases)**	***n*** **= 410** **(54 cases)**	
**Simplified FINDRISC**													
FPG-based diagnosis	E/O	0.43 [0.30–0.61]	1.16 [0.92–1.47]		1.11 [0.80–1.55]	0.84 [0.66–1.07]		0.63 [0.43–0.94]	1.00 [0.80–1.25]		1.13 [0.85–1.50]	0.81 [0.62–1.06]	
	C-Statistic	0.64 [0.53–0.76]	0.72 [0.66–0.78]	0.28	0.68 [0.58–0.78]	0.65 [0.58–0.72]	0.56	0.58 [0.44–0.72]	0.54 [0.47–0.62]	0.66	0.69 [0.60–0.77]	0.68 [0.61–0.75]	0.90
HbA1c-based diagnosis	E/O	0.57 [0.43–0.77]	0.86 [0.74–0.99]		1.21 [0.93–1.57]	0.66 [0.57–0.77]		0.67 [0.51–0.89]	0.82 [0.70–0.95]		0.97 [0.79–1.18]	0.68 [0.57–0.81]	
	C-Statistic	0.74 [0.65–0.83]	0.76 [0.72–0.80]	0.60	0.80 [0.73–0.86]	0.71 [0.6–0.76]	0.03	0.66 [0.58–0.75]	0.64 [0.58–0.69]	0.61	0.76 [0.71–0.81]	0.77 [0.72–0.82]	0.81
**Sample Size**		***n*** **= 261** **(15 cases)**	***n*** **= 676** **(34 cases)**		***n*** **= 447** **(12 cases)**	***n*** **= 490** **(37 cases)**		***n*** **= 477** **(13 cases)**	***n*** **= 460** **(36 cases)**		***n*** **= 527** **(27 cases)**	***n*** **= 410** **(22 cases)**	
**ADA**													
FPG-based diagnosis	E/O	2.58 [1.81–3.70]	2.82 [2.24–3.56]		2.39 [1.72–3.33]	2.75 [2.16–3.49]		3.15 [2.13–4.67]	2.43 [1.94–3.04]		2.88 [2.17–3.84]	2.65 [2.03–3.46]	
	C-Statistic	0.75 [0.63–0.87]	0.75 [0.68–0.83]	0.96	0.74 [0.64–0.83]	0.68 [0.59–0.77]	0.37	0.81 [0.73–0.89]	0.64 [0.55–0.74]	0.01	0.82 [0.74–0.89]	0.65 [0.54–0.76]	0.01
HbA1c-based diagnosis	E/O	2.70 [2.01–3.63]	1.73 [1.49-2.00]		2.39 [1.84–3.11]	1.73 [1.49–2.02]		2.81 [2.13–3.71]	1.59 [1.37–1.85]		2.12 [1.73–2.58]	1.79 [1.50–2.14]	
	C-Statistic	0.80 [0.70–0.90]	0.77 [0.72–0.82]	0.55	0.77 [0.69–0.86]	0.69 [0.62–0.76]	0.12	0.68 [0.58–0.78]	0.68 [0.62–0.75]	0.99	0.80 [0.74–0.86]	0.73 [0.66–0.80]	0.17
**Sample Size**		***n*** **= 261** **(15 cases)**	***n*** **= 676** **(34 cases)**		***n*** **= 447** **(12 cases)**	***n*** **= 490** **(37 cases)**		***n*** **= 477** **(13 cases)**	***n*** **= 460** **(36 cases)**		***n*** **= 527** **(27 cases)**	***n*** **= 410** **(22 cases)**	
**IRS**													
FPG-based diagnosis	E/O	0.68 [0.48–0.97]	0.98 [0.78–1.24]		1.03 [0.74–1.43]	0.79 [0.62–1.01]		0.99 [0.67–1.47]	0.80 [0.64-1.00]		0.98 [0.74–1.30]	0.83 [0.63–1.08]	
	C-Statistic	0.79 [0.69–0.89]	0.74 [0.65–0.82]	0.45	0.68 [0.56–0.80]	0.70 [0.61–0.79]	0.81	0.72 [0.60–0.84]	0.65 [0.56–0.75]	0.40	0.79 [0.70–0.87]	0.66 [0.55–0.78]	0.10
HbA1c-based diagnosis	E/O	1.08 [0.80–1.45]	0.92 [0.79–1.07]		1.42 [1.10–1.85]	0.78 [0.67–0.91]		1.25 [0.95–1.65]	0.83 [0.71–0.96]		1.09 [0.89–1.33]	0.86 [0.72–1.02]	
	C-Statistic	0.84 [0.75–0.92]	0.76 [0.71–0.82]	0.14	0.83 [0.76–0.89]	0.70 [0.64–0.77]	0.01	0.68 [0.60–0.77]	0.67 [0.61–0.74]	0.78	0.80 [0.74–0.86]	0.76 [0.69–0.83]	0.39

*Values [95% CI]; +P-values compare the C-statistic between stratified groups. FPG – fasting plasma glucose; HbA1c – glycated haemoglobin; E/O – expected/observed

Sample sizes differ for each risk prediction model, as participants with missing data were excluded per model

### Calibration

According to the E/O ratio, before recalibration, the ADRS, Simplified FINDRISC, and IRS models underestimated the prevalence of FPG- and HbA1c-based T2D, whereas the opposite was observed for the ADA risk score (Table 1). Recalibration improved the agreement between predicted and observed prevalent T2D rates for all models. Although the pattern of underestimation remained, the 95% CI of the E/O ratio for the ADRS, Simplified FINDRISC and IRS models all spanned 1, with the ADRS for FPG-based T2D diagnosis performing the best. The ADA risk model also improved upon recalibration but continued to overestimate T2D prevalence approximately 2-fold.

**Fig. 2:**
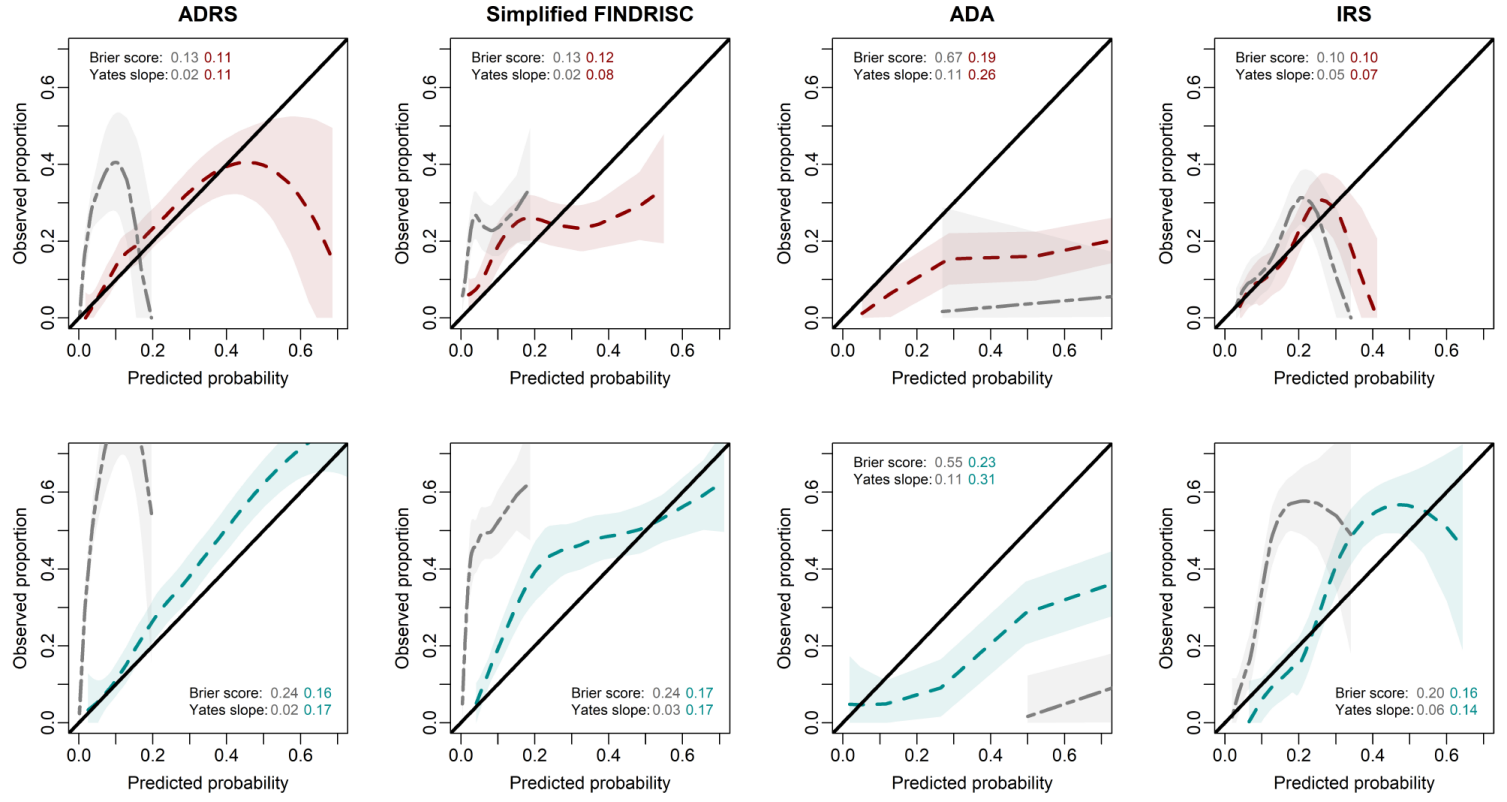
Calibration curves for each risk prediction model by FPG-diagnosis (red) and HbA1c-diagnosis (blue), and their respective Brier scores and Yates slopes. The grey curves represent the original calibration, and the coloured curves represent recalibration

The calibration curves are presented in Fig. 2. In agreement with the E/O data, there was a systematic risk overestimation across the continuum of predicted prevalence by the ADA model for both FPG and HbA1c as the method of diagnosis. When compared to FPG as diagnostic criteria, the ADRS and IRS had a selective upper stratum (when T2D probability was higher) risk overestimation. In contrast, the ADRS had a systematic underestimation across the continuum, and the IRS had a combination of overestimation in the lower strata and underestimation in the upper strata, using HbA1c as the method of diagnosis. The Simplified FINDRISC had a lower strata underestimation and an upper strata overestimation when using FPG as the method of diagnosis, whereas it had a systematic underestimation when HbA1c was used.

Regarding subgroup analysis (Table 2), the ADRS proved the most stable, with 95% CIs overlapping between each of the compared groups. The Simplified FINDRISC had two instances of non-overlapping prediction, and there was one instance for the ADA and IRS scores. Each of these three models also had one instance of very slight overlapping. The lack of overlap predominantly occurred with HbA1c-based diagnosis. The Simplified FINDRISC significantly overestimated FPG-based T2D prevalence in women compared to men. In contrast, although both were overestimated, the ADA risk score predicted much higher HbA1c-based T2D in men compared to women. Both the simplified FINDRISC and IRS predicted a higher HbA1c-based T2D prevalence for participants younger vs. older than 51 years. While both ratios remained higher than 1, the discrepancy in the expected vs. observed prevalence of HbA1c-based T2D was larger in participants with a lower compared to higher BMI for the ADA. Finally, the Simplified FINDRISC underestimated risk in urban vs. rural dwellers.

## Discussion

This study further validated the unpublished ADRS, the only risk prediction model developed in a sub-Saharan African population and compared its performance to that of other widely used T2D risk prediction models. To the authors’ knowledge, this is also the first study to evaluate whether the performance of risk prediction models differed when using HbA1c or FPG as the diagnostic measure of T2D. Though many T2D risk scores have been developed, only a few have been externally validated, usually in high-income countries [36]. Since prediction equations developed in other population groups that include non-invasive variables only, have performed poorly in African cohorts [14, 16], it is important to identify a suitable risk prediction model for use in individuals of African descent. The external validation study by Masconi et al. [14] also highlighted the need to improve screening tools for use in the South African setting. In terms of a public health approach, a non-invasive risk score can be used to identify individuals at risk of prevalent undiagnosed T2D who should undergo further biochemical testing [37]. This will ensure the allocation of scarce resources to those at the highest risk to prevent the progression of T2D and associated complications in a cost-effective manner. An ideal screening tool should thus be easy to use, non-invasive, free, and easily accessible to clinicians, public health workers, researchers, and individuals to assess their level of risk of having T2D [11, 15].

### Overall performance

Almost all participants with T2D reported not being previously diagnosed with T2D, which agrees with the high prevalence of undiagnosed T2D in SA [2]. In the SA-NW-PURE sample, the ADA overestimated risk, and the remaining prediction models performed relatively comparably. Except for the ADA, the other models had an overall modest-to-acceptable discriminatory ability to predict prevalent undiagnosed T2D, using both FPG and HbA1c as diagnostic criteria. However, the ADRS and IRS performed marginally best when FPG and HbA1c, respectively, were used, taking C-statistics, E/O rates, sensitivity, and specificity into account. The age cut-off in the population used to develop the Simplified FINDRISC was much higher than the other three models, and FINDRISC only included the use of blood pressure medication (and not blood pressure itself) to determine hypertensive status (thus assuming that all people with hypertension were receiving treatment), which could have negatively impacted its performance in this population.

The calibration for all models was improved through simple intercept adjustment, and the assessment of recalibration was better when FPG was used. After recalibration, the ADRS had a near-perfect E/O ratio when FPG was used for diagnosis, and the calibration curve was close to the curve of perfect calibration, with slight overestimation in the upper strata only. Furthermore, the ADRS performed equally well in all subgroups, further supporting its generalisability. This is not unexpected as the three variables that the ADRS uses; age, waist circumference, and hypertension, are known to be strong predictors of T2D [38–42] and commonly feature in risk prediction models. The fact that the recalibration had the smallest effect on the IRS suggests that the baseline prevalence in the population in which the IRS was developed was similar to that of the SA-NW-PURE study. In contrast, the baseline risk in the population in which the ADA model was developed was much higher, and while recalibration was slightly successful in reducing the degree of risk overestimation, this remained unacceptably high.

The number of variables used in the equations does not seem to influence the predictive performance of risk scores [12]. However, the more variables the model uses, the higher the chance of participant exclusion due to missing data. This is seen with the ADA, which had the largest number of variables (*n* = 6) and the smallest sample size (*n* = 420). Though the ADRS had the fewest variables (*n* = 3), the Simplified FINDRISC, with five variables, had the largest sample size (*n* = 727).

Considering the aforementioned results, the ADRS is recommended for use in this Tswana-speaking, Black South African population. Though the IRS performed marginally better when HbA1c was used, it includes family history of diabetes and level of physical activity as variables, which may result in missing data or incorrect information used for risk calculation. This is particularly relevant in older populations, and those with a relatively low level of education, poor access to health care, and unhealthy lifestyles [43]. The ADRS includes age, waist circumference and hypertensive status as variables, all of which are easy to obtain. Fewer variables reduce the risk of missing data and the impact this has on the performance of a model. The ADRS has been externally validated by Mayige [17] in an urban Black population in Cape Town (CRIBSA study population), and a rural Black population in Kwa-Zulu Natal, and it also performed well in these populations. Although ethnicity was not genetically determined in this or the external validation studies by Mayige [17], individuals in the North-West Province, Cape Town and Kwa-Zulu Natal typically represent different ethnicities, and these results, therefore, suggest that the ADRS can successfully be used in different ethnic groups in South Africa.

### Performance according to subgroups

The models’ performance varied in the major subgroups, where they generally performed better in women, those who were younger, and those with a lower BMI. Discrimination varied in the subgroups from poor to good. Women accounted for 70% of the sample of this study. Certain risk factors for T2D may affect men and women differently, such as distinct hormonal profiles in women that influence their risk [44], or sex differences in health-related behaviour, such as smoking, excessive drinking, and unhealthy dietary habits, that can alter diabetes risk [45]. Men are also more insulin resistant than women and have greater central and hepatic fat compared to women [46, 47]. If the model has been developed using risk factors that are more common in one sex, it may not accurately assess risk in the other. For example, Mayige [17] indicated that women were over-represented in the studies used to derive and validate the ADRS. Moreover, T2D is strongly associated with obesity [10], and in individuals with lower BMI levels, other risk factors for T2D may become more significant, allowing the model to detect and weight these factors more effectively. It is thus important that individuals with a range of BMI levels are included in the development of a risk prediction model, so the diversity can improve the model’s ability to evaluate a range of risk factors and their interactions.

The median age of the SA-NW-PURE population was 51 years; thus, a large proportion of the study population was at risk of T2D, as age is a key risk factor for developing T2D [10]. Ideally, screening for T2D should begin from 35 years for all people and should take place in adults of all ages if they present with overweight or obesity and one or more risk factors for T2D [10, 48]. Younger individuals usually have a lower baseline risk of developing T2D, and they may have fewer risk factors that trigger a model’s criteria designed to detect individuals at elevated risk. The underestimation of risk in urban dwellers could result from the model inadequately accounting for the nutrition transition that occurs with urbanisation and the resultant increased risk of T2D [49].

### Performance according to the method of T2D diagnosis

This study has shown that the models performed comparably, whether FPG or HbA1c was used. This has important implications for future screening of undiagnosed T2D. HbA1c may be more convenient to obtain when conducting epidemiological research as it doesn’t require fasting. It has long been accepted that FPG can be used to diagnose diabetes [50]. Relative to FPG, HbA1c has only been accepted as a screening and diagnostic test more recently, after much debate. Other than glycaemia, several factors can affect HbA1c levels [51]. For example, HbA1c can portray falsely high glucose levels when red blood cells have an increased lifespan and/or reduced turnover, as is found in untreated iron deficiency and certain haemoglobin variants [52]. Haemoglobinopathies have been found to be more prevalent in some LMICs [1].

Furthermore, it has been reported that HbA1c and FPG have slight differences in identifying different groups of people with diabetes [53, 54]. HbA1c levels have also been demonstrated to differ between races, potentially affecting the T2D prevalence when assessed by HbA1c and FPG [53, 55]. Furthermore, it has recently been suggested by Chivese et al. [56] that a different HbA1c cut-off may need to be applied for individuals of African descent since the T2D HbA1c cut-off of 6.5% missed up to 42% and 35% of people with diabetes identified by OGTT and FPG, respectively. This does not imply that glucose-based measures are more accurate in classifying T2D; however, the discrepancy between tests may result in differing estimates of the prevalence of T2D [56]. Though the performance of the risk prediction models in this study was comparable with both methods of diagnosis, they tended to underestimate risk when HbA1c was used. This finding supports the suggestion of a lower HbA1c cut-off for individuals of African descent.

### Strengths and limitations

This study has assisted with the generalisability of the ADRS in Black South Africans, who account for the greatest portion of people in South Africa [57]. As prediction models commonly perform poorly in new populations compared to the development population [28], investigators tend to reject existing models and develop or fit a new one. This results in the loss of previous scientific information and creates confusion amongst healthcare professionals as to which model to use. Better practice involves updating and recalibrating existing prediction models to the population at hand [28]. This results in combined information from the original model and new individuals [58–60]. The combination of information may improve the generalisability of the updated model to other population groups [28]. A potential limitation of the study was the large amount of missing data for some variables, reducing the sample size available for validating each risk score. This could have negatively influenced their performance. Limited information was available for family history of T2D, which resulted in very small sample sizes for the ADA and IRS. The oral glucose tolerance test is considered to be the gold standard for diagnosing diabetes, and not having this measurement in the SA-NW-PURE dataset could therefore be considered a limitation of the study.

## Conclusion

Widespread laboratory measurements are not financially feasible in resource-scarce settings such as South Africa. Ideally, scarce resources should be allocated to those at highest risk of developing or having undiagnosed T2D. A two-step approach can thus be used, where a risk prediction model comprised of non-invasive variables in the form of a questionnaire can guide healthcare professionals on whether a diagnostic test should be performed. While no model significantly outperformed others enough to be uniquely selected for routine risk stratification use, based on the ease of use and the performance, it is recommended that the ADRS be used to screen for T2D in the Black population groups in South Africa. It can also be used as a health promotion tool when used as a self-completion questionnaire. Furthermore, using HbA1c as a means of diagnosis resulted in comparable performance with FPG. Therefore, further validation of risk prediction models can potentially make use of HbA1c as a method of diagnosis, given its convenience as it does not require fasting.

## Electronic supplementary material

Below is the link to the electronic supplementary material.

Supplementary Material 1

## Data Availability

The datasets used and/or analysed during the current study are available from the corresponding author on reasonable request.

## Abbreviations

ADA: American Diabetes Association
ADRS: African Diabetes Risk Score
BMI: Body mass index
E/O: Expected and observed rates
FINDRISC: Finnish Diabetes Risk Score
rRNA: Ribosomal RNA
FPG: Fasting Plasma Glucose
HbA1c: Glycated haemoglobin
AUROC: Area Under The Receiver Operating Characteristic Curve
IRS: Indian Risk Score
ISAK: International Standards of Anthropometric Assessment
IGF-1: Insulin-Like Growth Factor-1
LMICs: Low- and Middle-Income Countries
NWU: North West University
ROC: Receiver Operating Characteristic
SA-NW-PURE: South African North-West Province arm of the Prospective Rural and Urban Epidemiology
T2D: Type 2 Diabetes
ZAR: South African rand
