# Supplementary material for: HbA1c comparable to fasting glucose in the external validation of the African Diabetes Risk Score and other established risk prediction models in Black South Africans
Source: BMC Endocr Disord. 2024 Oct 10;24:213. doi: 10.1186/s12902-024-01735-w (PMC11465613; doi:10.1186/s12902-024-01735-w)
Supplement: Supplementary file 1 — Supplementary Material 1 [file 12902_2024_1735_MOESM1_ESM.docx]

**SUPPLEMENT**


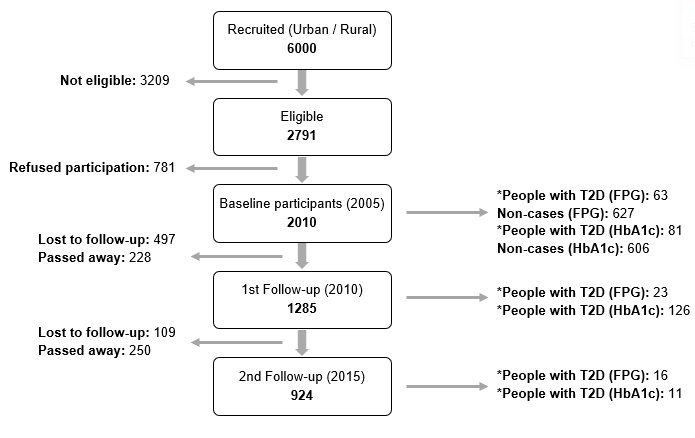


Supplemental Figure 1: Recruitment strategy (Total number of participants with T2D according to FPG = 102 (14%) & HbA1c = 218 (26%)). *Indicates that only newly diagnosed individuals were considered at the respective time points.

**Supplemental Table 1: Characteristics of the respective risk prediction models and how these were incorporated into the SA-NW-PURE study population.**

| **Characteristic** | **ADRS** | **Simplified FINDRISC** | **ADA** | **IRS** | **SA-NW-PURE** |
| --- | --- | --- | --- | --- | --- |
| Characteristics of the populations in which the scores were developed | | | | | |
| Authors | Mayige | Bergmann *et al.* | Bang *et al.* | Ramachandran *et al.* | - |
| Year published | 2014 (unpublished) | 2007 | 2009 | 2005 |  |
| Country in which score was developed | Multi-country | Germany | United States of America | India | - |
| Sample size | 5,411 | 526 | 5,258 | 4,993 | 937 |
| Design | Cross-sectional | Prospective | Cross-sectional | Cross-sectional | Repeated cross-sectional |
| Age (years) | ≥ 15  Tanzania: 24 – 64 years  Guinea: 25 – 64 years  Senegal: ≥ 15 years | 41 – 79 | 20 | 20 | > 30 |
| Population | Black (Tanzanian, Guinean and Senegalese) | European descent (Finland) | Multiracial (White, Hispanic, Black; USA) | Indian | Black (African descent; South Africa) |
| Definition of diabetes | FPG 7.0 mmol/l | FPG ≥ 7.0 mmol/l, or 2-hour glucose ≥ 11.1 mmol/l | FPG 7.0 mmol/L | FPG 7.0 mmol/l, or 2-hour glucose 11.1 mmol/l, or HbA1c 6.5% | FPG 7.0 mmol/l, or HbA1c 6.5% |
| Variables and equations of the respective scores | | | | | |
| Age (years) | Linear (years) | +0: ≤ 45  +0.628: 45–54  +0.892: >55 | +0: < 40  +1: 40–49  +2: 50–59  +3: ³ 60) | +1.05: 30 – 44  +1.75: 45 – 59  +1.93: > 59 | Data available |
| Sex | Not included | Not included | Men  Women | Not included | Data available |
| BMI (kg/m^2^) | Not included | +0: ≤ 25  +0.165: 25–29.9  +1.096: >30 | +1: 25≤ to <30  +2: 30 ≤ to <40  +3: ≥40 | +0: < 25  +0.729: 25 | Data available |
| Waist circumference (cm) | Linear (cm) | Sex-specific (F/M)  +0: < 80 or 94  +0.857: 80–88 or 94–102  +1.350: ≥ 88 or 102 | Sex-specific (F/M)  +1: 80 ≤ to < 89 or 94 ≤ to < 102  +2: 89 ≤ to < 124 or 102 ≤ to < 127  +3: ≥ 124 or ≥ 127 | +0.528: 80 (women)  +0.528: 85 (men) | Data available |
| History of Diabetes | Not included | Have you ever been told by a health-care professional that you have diabetes or latent diabetes?  No/latent diabetes/diabetes | Not included | Not included | Data available |
| Family history | Not included | Not included | Mother / Father  Brother / Sister | Yes  No | Data available |
| Physical activity | Not included | Not included | No  Yes | No  Yes | Data available |
| Hypertension diagnosis (mmHg) | SBP ≥ 140  DBP ≥ 90 | Not included | SBP ≥ 140  DBP ≥ 90 | Not included | SBP & DBP available and coded to define hypertension according to the respective risk models |
| Use of HTN drugs (question) | No  Yes | No  Yes | No  Yes | Not included | No  Yes |
| Linear predictor | X = [(-11.012+ 0.045(age)+ 0.048 (waist circumference) + 0.649 (hypertension)] | X = -5.514 + age + BMI + waist circumference + 0.711 (if prescribed antihypertensive medication) + 2.139 (if a history of high blood glucose, not relevant for SA-NW-PURE validation) | X = age + (men)*1 + (family history of diabetes)*1 + (history of hypertension)*1 + BMI/waist circumference - exercise*1. | X = -4.96 + age + 0.677(positive family history) + BMI + waist circumference + 0.437(sedentary & light physical activity) | - |

FPG – fasting plasma glucose; HbA1c – glycated haemoglobin; BMI – body mass index; M – male; F – female. The Simplified FINDRISC and IRS models included both BMI and waist circumference, whereas the ADA model included either BMI or waist circumference to classify obesity.

**Supplemental Table 2: FPG- vs. HbA1c-based T2D frequency in the SA-NW-PURE cohort.**

| **Cohort** | **Number of Participants (%)** |
| --- | --- |
| FPG classification (≥ 7 mmol/L) |  |
| Participants with T2D | 102 (14%) |
| Participants without T2D | 627 (86%) |
| HbA1c classification (≥ 6.5%) |  |
| Participants with T2D | 218 (26%) |
| Participants without T2D | 606 (74%) |

**Supplemental Table 3: Descriptive statistics of the study sample.**

| **Characteristic** | **Total Sample**  (n = 937) | **Cases According  to FPG**  (n = 102) | **Non-Cases According to FPG**  (n = 627) | **Cases According  to HbA1c**  (n = 218) | **Non-Cases According to HbA1c**  (n = 606) |
| --- | --- | --- | --- | --- | --- |
| Women n (%) | 676 (72.1) | 71 (69.6) | 440 (70.2) | 174 (79.8) | 410 (67.7) |
| Age (years) | 50.5 [43.8, 58.0] | 56.0 [46.2, 63.1] | 48.4 [42.4, 55.0] | 56.4 [49.7, 62.8] | 48.2 [42.2, 55.0] |
| Rural living status n (%) | 527 (56.2) | 47 (46.1) | 376 (60.0) | 97 (44.5) | 368 (60.7) |
| Hypertensive n (%) | 519 (55.4) | 77 (75.5) | 311 (49.6) | 149 (68.3) | 295 (48.7) |
| Systolic blood pressure (mmHg) | 131 [117, 146] | 139 [125, 154] | 128 [115, 145] | 134 [120, 150] | 128 [115, 145] |
| Diastolic blood pressure (mmHg) | 87.0 [79.0, 96.0] | 91.0 [82.8, 98.2] | 86.0 [78.0, 96.0] | 89.0 [81.0, 96.5] | 86.0 [78.0, 96.0] |
| Waist circumference (cm) | 80.8 [71.6, 92.2] | 92.0 [82.8, 99.6] | 76.8 [70.4, 88.0] | 91.5 [82.9, 100.5] | 76.1 [70.0, 85.7] |
| Body mass index (kg/m^2^) | 24.9 [20.3, 31.0] | 28.8 [25.1, 32.7] | 23.3 [19.8, 29.1] | 30.0 [25.6, 35.1] | 22.7 [19.6, 27.7] |
| Normal weight [BMI < 25 kg/m^2^] n (%) | 477 (50.9) | 25 (24.8) | 374 (59.6) | 50 (22.9) | 387 (63.9) |
| Overweight [BMI 25–29.9 kg/m^2^] n (%) | 197 (21.0) | 35 (34.7) | 112 (17.8) | 59 (27.1) | 103 (17.0) |
| Obese [BMI ≥ 30 kg/m^2^] n (%) | 263 (28.1) | 41 (40.6) | 142 (22.6) | 109 (50.0) | 116 (19.1) |
| Weighted physical activity | 2.9 [2.6, 3.2] | 2.7 [2.5, 3.1] | 2.9 [2.6, 3.3] | 2.9 [2.6, 3.1] | 2.9 [2.6, 3.3] |
| Fasting glucose (mmol/L) | 5.0 [4.5, 5.6] | 7.8 [7.2, 9.6] | 4.8 [4.4, 5.2] | 5.6 [5.1, 6.8] | 4.8 [4.4, 5.2] |
| HbA1c (%) | 5.7 [5.4, 6.4] | 6.7 [5.9, 8.4] | 5.5 [5.3, 5.8] | 6.8 [6.6, 7.2] | 5.5 [5.2, 5.7] |
| Ever used tobacco n (%) | 493 (52.6) | 50 (49.0) | 338 (53.9) | 109 (50.0) | 336 (55.4) |
| Ever alcohol consumer n (%) | 341 (36.4) | 38 (37.3) | 241 (38.4) | 62 (28.4) | 246 (40.6) |
| Self-reported History of T2D: Yes n (%)* | 34 (3.63) | 17 (16.7) | 3 (0.48) | 6 (2.75) | 3 (0.50) |
| Family history of T2D: Yes n (%) | 70 (7.47) | 7 (6.86) | 47 (7.50) | 17 (7.80) | 43 (7.10) |

Continuous variables are reported as median [Q1, Q3]. BMI – body mass index; T2D – type 2 diabetes.

*Question: Do you have diabetes? Yes/No. All participants included in this study were newly diagnosed with T2D, or T2D-free, according to FPG and HbA1c.
